# Connecting knowledge with action for health equity: a critical interpretive synthesis of promising practices

**DOI:** 10.1186/s12939-019-1108-x

**Published:** 2019-12-26

**Authors:** Katrina M. Plamondon, C. Susana Caxaj, Ian D. Graham, Joan L. Bottorff

**Affiliations:** 1 School of Nursing, The University of British Columbia, 1147 Research Rd., ART 360, Kelowna, BC V1V 1V7 Canada; 2 School of Nursing, Western University, London, Canada; 3 School of Epidemiology & Public Health, University of Ottawa, Ottawa, Canada

## Abstract

Connecting knowledge with action (KWA) for health equity involves interventions that can redistribute power and resources at local, national, and global levels. Although there is ample and compelling evidence on the nature, distribution, and impact of health inequities, advancing health equity is inhibited by policy arenas shaped by colonial legacies and neoliberal ideology. Effective progress toward health equity requires attention to evidence that can promote the kind of socio-political restructuring needed to address root causes of health inequities. In this critical interpretive synthesis, results of a recent scoping review were broadened to identify evidence-informed promising practices for KWA for health equity. Following screening procedures, 10 literature reviews and 22 research studies were included in the synthesis. Analysis involved repeated readings of these 32 articles to extract descriptive data, assess clarity and quality, and identify promising practices. Four distinct kinds of promising practices for connecting KWA for health equity were identified and included: ways of structuring systems, ways of working together, and ways of doing research and ways of doing knowledge translation. Our synthesis reveals that advancing health equity requires greater awareness, dialogue, and action that aligns with the what is known about the causes of health inequities. By critically reflecting on dominant discourses and assumptions, and mobilizing political will from a more informed and transparent democratic exercise, knowledge to action for health equity can be achieved.

## Background

Health inequities are systematic differences in health at local, regional, national, and global levels [1–3], caused by the unfair distribution of resources, wealth, and power in society [4]. They are unjust [5] and actionable [6, 7]. Despite the strength of evidence and availability of feasible policy recommendations [8–10], connecting knowledge with action for health equity remains limited. The problem is not one of inadequate evidence, but rather “inadequate or ineffective knowledge translation” combined with discordant ideologies ([11] p. 54) and epistemologies [12]. These barriers can lead to a focus on relieving the downstream symptoms, rather than interrupting the known causes of health inequities [13, 14]. Confronting this problem requires evidence-informed strategies that support people to move toward better alignment between good health equity intentions and *evidence-informed* action.

Grounded in an assumption that advancing health equity involves interrupting routine practices that maintain unfair distributions of power and resources in society, the question guiding this critical interpretive synthesis was: *what promising practices for connecting KWA for health equity are evident in the literature?* Practices were understood as habitual, customary actions objectively and subjectively constructed through the routines people carry out in social contexts [15]. *Promising* practices were understood as those for which there was some empirical evidence to suggest they can inspire productive action toward a desired outcome. To the best of our knowledge, this is the first review to critically examine promising and empirically-derived strategies for advancing productive action on the root causes of health inequities.

## Methods

Critical interpretive synthesis (CIS) involves systematic analysis of a nebulous and complex literature using exploratory, rather than hypothetical, research questions [16]. It relies on iterative qualitative analysis and synthesis of emerging findings. It was shaped by theoretical foundations of critical pedagogy [16, 17], critically reflective inquiry [18], and relational theory [19]. Our positionalities and experiences as researchers deeply immersed in the field of health equity and knowledge translation (KT) were inextricable from our analysis. We adopted a critical lens throughout our synthesis, with an explicit assumption that power and history shape knowledge claims [17, 20] and that health inequities are human-caused, unacceptable, and actionable.

Selecting articles for this study involved applying new inclusion and exclusion criteria to a body of literature identified for a scoping review [14] (Table 1). Empirical studies and literature reviews published between 2008 and 2016 were included if authors framed health inequities as having known causes.

**Table 1:** Search Terms, Inclusion, and Exclusion Criteria

Search Terms	
Connecting knowledge with action	(knowledge OR evidence OR research OR guideline*) near to (utiliz* OR utilis* OR uptake OR transfer OR translat* OR transmit* OR transmission OR effectiveness OR populari* OR exchange OR synthes* OR transform* OR linkage* OR disseminat* OR implement* OR exchange)
Focus on health equity	(health OR social) near to (inequit* or equit* or equal* or unequal* or justice* or injustice* or disparit*)
Inclusion and Exclusion Criteria	
Inclusion Criteria	1. Empirical studies (research, systematic literature reviews, syntheses) 2. Published post-publication of Commission on Social Determinants of Health (2008–2016) 3. Implicit or explicit knowledge-to-action intention 4. Orientation to addressing health inequities 5. Problematized health inequities by citing evidence of socioeconomic, historical, political roots and/or the maldistribution of resources, money, and power (e.g., CSDH) 6. Study is clearly positioned in a productive orientation toward root causes of inequities (i.e., seeks to acknowledge, illuminate, or interrupt root causes).
Exclusion Criteria	1. Naturalized systematic differences in health and health outcomes 2. Did not discuss role of power and privilege in health inequities 3. Orientations toward root causes were not productive (disregard, distract, discredit) 4. Did not present an argument about how to connect knowledge to action for health equity 5. Did not present results (e.g., study protocol)

Synthesis involved repeated readings of included articles using constant comparative methods [21]. Nvivo 11 for Mac and Microsoft Excel supported data management and organization. In the first reading, descriptive and bibliometric data were extracted (Table 2) and articles were assessed for clarity and quality [22] (Table 3). In subsequent readings, we considered the explicit and implicit assumptions embedded in language, study designs, analytical approaches, and claims. Attention was placed both on how the researchers approached and framed their work and on their reported results. In the final reading, articles were reviewed for gaps, silences, and omissions.

**Table 2:** Data Extraction Elements

Element	Description
Bibliometric data	Author, year, source journal, discipline, location
Study purpose	Direct quotation of statement of aims, goal, or purpose
Methods	Methodology and data generating approaches Assessment of the clarity and quality of research methods
Practices tested or derived	Specific actions, processes, ways of working that are either tested or emerge from the study findings
Evidence for promising practices	Arguments, research findings, and claims made by authors about strategies, facilitators or barriers, or approaches that demonstrate some degree of promise for connecting KWA for health equity

**Table 3:** Assessment Criteria and Scores for Clarity and Quality of Research Methods^a^

Prompts	Absent, unidentifiable (Score = 0)	Not Clear or Vague, partial (Score = 1)	Clear, well described (Score = 2)
(AO) Are the aims and objectives of the research clearly stated?	No clear statement of aims and objectives.	Aims and objectives implied, but difficult to discern.	Aims and objectives explicitly stated and easily identifiable.
(DES) Is the research design clearly specified and appropriate for the aims and objectives of the research?	Study design does not align with aims and objectives. and/or Little to no description of methodological approach provided.	Study design somewhat aligned with aims and objectives. and/or Some description of methodology provided, but with gaps or use of generic language used to describe methodology (e.g., ‘qualitative’).	Study design aligns with aims and objectives. and Methodological approach and theoretical foundation clearly described.
(MET) Do the researchers provide a clear account of the process by which their findings were produced?	No clear description of study process of data generation, making it impossible to replicate study.	Data generation and analytical processes vaguely described— would be difficult to replicate study.	Methods and analytical process clearly described, consistent with methodological approach and theoretical foundation— would be possible to replicate study.
(D) Do the researchers display enough data to support their interpretations and conclusions?	Insufficient data presented to support authors’ claims.	Difficult to discern if data is sufficient to support authors’ claims.	Data presented is compelling and clearly supports authors’ claims.
(AN) Is the method of analysis appropriate and adequately explicated?	Analytical processes inadequate or absent; not clearly or coherently linked to conclusions.	Analytical processes vaguely described; difficult to determine coherency with study design and findings.	Analytical process well described, coherent with methodology, and logically connected to authors’ conclusions.

^a^Criteria derived from Dixon-Woods et al. (2006)

## Results

**Fig. 1:**
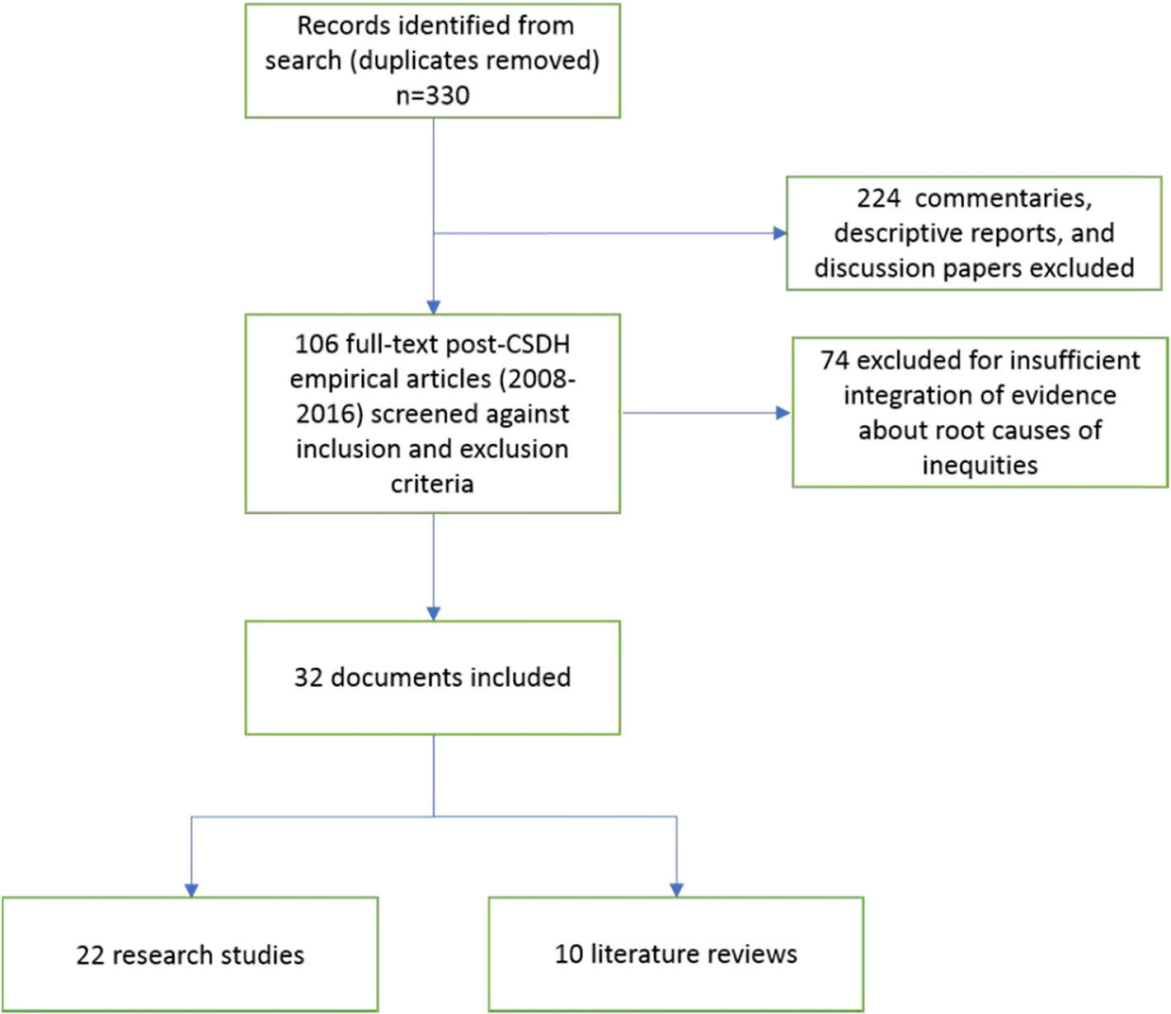
Search and selection results for CIS

Of 330 articles screened against inclusion and exclusion criteria, 10 literature reviews, 4 of which were scoping reviews [23–26], three syntheses [27–29], one realist review [30], and two rapid reviews [31, 32] were included. A total of 22 research studies were also included:7 case studies [33–37] or case series [38, 39]; 6 were described as ‘qualitative’ [40–45]; 3 involved policy [46, 47] or discourse analysis [48]; 1 was survey-based [49]; 2 were described as ‘mapping’ [50, 51]; 1 was mixed methods [52]; and 2 described as some form of action research [53, 54] (Fig 1). Overall, studies in this review were of relatively high clarity and quality. All but two of the articles’ [29, 51] primary authors were from the health professions or health sciences, all were published in health-related journals, and all involved health professions or health sciences in the authorship team. A detailed overview of the descriptive data extracted and assessment of article clarity and quality are available in Additional file 1: Table S1 (literature reviews) and Additional file 2: Table S2 (research studies).

### Four promising practices of connecting KWA for health equity

Four groups of promising practices for connecting KWA for health equity were identified, related to: (re) structuring systems, working relationally, doing research, and carrying out KT.

#### Promising practices for (re) structuring systems

Promising practices for structuring systems to support KWA for health equity included integrating equity-oriented governance mechanisms, embedding a policy of health equity at systems-levels, and strategically navigating bureaucratic hierarchies (Additional file 3: Table S3). Among studies reviewed, researchers consistently reported that the architecture of systems influenced the focus and energy devoted to the social determinants of health or health equity work. Cultivating clear governance mechanisms was identified as a promising structural intervention to foster organizational enviornments conducive to health equity action [23, 37, 39, 44, 47, 50]. Several authors identified the importance of savvy governance structures that recognized the hierarchical nature of bureaucracies and the need for clearly aligned health equity agendas with institutional mechanisms to enable action [23, 26, 27, 32, 37, 42, 45, 48]. Each of these studies pointed to the political vulnerability of health equity agendas, problematizing the broader socio-political hierarchies within which healthcare systems operate.

These hierarchies were directly linked to social power. The influence of social positioning and power on the ability of community health workers to fulfil their roles in health equity work was, for example, discussed by Labonté and colleagues [39]. McPherson et al. [37] also examined social power, identifying the impact of senior and middle management support on a health equity agenda and the role of a dedicated nursing role focused on social determinants of health in a qualitative case study. These researchers found that deploying *social determinants of health nurses* within the healthcare system was a key determinant of the efficacy and direction of their health equity work. Despite provincial mandates for nurses to be involved in reporting about the impact of their roles however, some participants reported that they did not have a voice in shaping what or how health equity work was reported.

These findings were echoed in a scoping review on public health advocacy [24], where authors reviewed research in which public health nurses reported feeling powerless because of administrative constraints, particularly because of constraints they experienced as a result of excessive workloads, apathy, and incoherence between the downstream focus that tends to dominate healthcare and the upstream demands of health equity work [24]. In both these examples, authors noted that vulnerability to bureaucratic culture, hierarchy, and norms reinforce the need for strategic positioning and support of health-equity-oriented roles. Two studies found that heath professionals with responsibility for health equity were restricted by the way their workplace was structured and governed, particularly when evaluated within an organizational culture resistant to change [37, 39]. Collectively, these results illuminate how hierarchy and social power can generate or restrict permission to do structurally-oriented health equity work.

#### Working relationally as a promising practice

Promising practices individuals and organizations can use for working relationally to achieve health equity goals focused on fostering inclusion (who is involved) and connectedness (how they are involved), and mitigating power imbalances (Additional file 4: Table S4). Inclusion was often described as a means for overcoming power imbalances in society and about extending the scope of imagination for who to engage in health equity work. Fostering inclusion and connectedness were both described as more effective when health equity work focused on upstream, structural determinants of health and explicitly included attention to the role of power in shaping why, who, and how particular groups are included or excluded [52, 53]. In two studies, authors concluded that participation in health equity research or KT is not de facto inclusive while maintaining that inclusivity was necessary to advance equity [35, 39]. Several articles, for example, explored the importance of inviting non-scientific and non-health actors into health equity work because it involves transforming social and political environments that rely on political will and public sentiment [40, 42, 44–46].

Fostering connections was also described as a means of illuminating relationships between causes and outcomes of inequities, particularly when health equity work involved some form of policy influence. Several studies indicated, for example, that creating an environment of pressure on decision makers and power-holders was important [28], with public engagement as a key mechanism to do so [24, 27, 32, 50]. In one study, illuminating the connection between public policy and narratives of ‘worthiness’ (i.e., what problems and people deserve public attention) was identified as important, particularly because it directed health equity efforts at appealing to public sentiment and political will [43]. Benefits of fostering inclusion and connectedness included improving sustainability [24, 28, 33, 41]; enhancing collective appreciation for the contributions of different domains of research [25, 35, 50]; enabling context-responsiveness [25, 37]; and enhancing capacity to navigate complexity [23, 25, 26, 31, 32, 35, 39, 40, 50, 51].

Practices of fostering inclusion and connectedness were inextricable from practices of mitigating power imbalances. Two articles described paying attention to power dynamics within their own research teams: one purposively sought to overcome representational and power inequities in the research process itself [39, 53]. Some authors discussed the need for greater awareness and mitigation of power imbalances in health equity work [26, 45, 54], and particularly in how it shapes the distribution of wealth in society [37]. Evidence also supported the benefits of allyship, where people leveraged their power and resources to contribute to more equitable processes or outcomes of research [30, 39, 41, 53]. For example, researchers described leveraging their positional authority or access to research grants [39, 53] to redistribute resources in ways that balanced historical inequities.

#### Promising practices for doing research

Promising research practices involved embracing complexity in health equity work, using dialogic-relational methods, and ameliorating data gaps (Additional file 5: Table S5). Complexity was acknowledged as a critical consideration in virtually all of the articles reviewed, with authors speaking to its importance across the research process from problem definition, to identifying targets of intervention and navigating research partnerships. In one scoping review [23], authors identified risks of oversimplification when populations are defined as the problem rather than the structural contexts that led to their disproportionate burden of health inequities. In another example—an international qualitative study—context sensitivity was described as acknowledging history and understanding the complex landscape well enough to foster inclusion [41]. Among the strategies authors used to embrace complexity were dialogic and relational approaches to research [25, 34, 50], particularly because such approaches take “better account of the complexities inherent in health problems, particularly health inequities” ([29], p., 2089). Researchers using these dialogic approaches often described a commitment to engaging diverse perspectives, which was identified as instrumental to navigating complexity in relationship with communities that ultimately open possibilities for political action.

Data systems and data handling were frequently discussed as mechanisms that either reinforced or challenged the status quo. In particular, authors noted that health research designs often rely on the use of downstream indicators to define and monitor aggregate populations; yet, the use of such indicators poses problems for generating evidence about progress on the broader social and structural determinants of health inequities [36, 38, 41, 46, 50–52, 54]. Further, researchers problematized insufficiencies in standards of data aggregation and in the adequacy of indicators. In several studies, artificial inflation of health equity gains was ascribed to data aggregation [36, 51] that can mask or entrench inequities by extending population boundaries that misrepresent social stratification that leads to inequities [36, 38, 46, 52, 54]. For example, the use of normative categories can play a role in masking inequities through binaries that exclude non-cisgender individuals [36, 52, 54]. Efforts to monitor and report on health equity often rely on administrative data that reflects aggregated service usage (e.g., geographic access) statistics and other individualized biomedical measures [48], few of which actually measure health improvement or the distribution of power, resources, or wealth in society. These authors and others called for broadened surveillance systems [39, 41, 50] and re-imagined indicators, mechanisms, and methodologies to enable the longitudinal study of upstream interventions that address causes of inequity [37, 41, 48, 50, 52, 54].

#### Promising practices for knowledge translation

Promising practices in KT for health equity validated the use integrated knowledge translation (or doing research with people who use research, throughout the research and knowledge translation process). Additionally, the use of creative strategies to complement numeric data with stories about the lived experience of inequities (Additional file 6: Table S6) was also found to be promising. In one review of KT frameworks [25], researchers concluded that the most equity-responsive frameworks were applied, impact focused, prioritized engagement and were trust-building, emphasized context, built in mechanisms for addressing issues of power, and contained inclusive definitions of the ‘knowledge’ of KWA. Their discussion pointed to the importance integrated approaches to KT, where researchers work in responsive partnership with the people and organizations or settings where the research is intended to inform action. They found careful attention to who is engaged and targeted to be particularly important. Inclusion was recognized as important to informing action in several other studies, with particular attention on the importance of engaging the public as a KT target [24, 26, 34, 50]. Others affirmed this importance in their calls for public health *advocacy* that shifts attention away from individual and behavioural interventions toward broader conceptualizations of public issues that invite community mobilization [28, 42, 43].

There was also evidence to support developing a curated or storied approach to presenting evidence, and packaging it in creative ways that present a concise and compelling story [28] alongside feasible policy options [33, 40]. One meta-synthesis of policy research supported a similar finding, identifying as beneficial the presentation of timely, real-life data that blends numbers with human experiences and is tailored to different decision needs [34]. Authors argued for KT approaches and products that inspire decision makers [55] and health professionals [28, 34, 44–46, 52] by evoking empathy and sparking imagination for more compassionate responses that can mobilize human agency to overcome health inequities.

### Gaps in the literature

Although health equity work logically involves health research, the nature of health inequities suggests that it is it is necessary to embrace cross-sectoral and multi-disciplinary approaches. This body of literature, however, demonstrated little engagement outside the health sector. And although many authors specifically emphasized the importance of clarity, transparency, and examination of assumptions and explicit power analysis in health equity work [28, 29, 36, 42, 44, 51, 54], few [25, 48, 52] demonstrated analysis of epistemological underpinnings, methodological foundations, or power. Another gap observed in this literature was the absence of attention on or calls for sensitizing political economy actors (e.g., business, economics, management, political sciences) and other non-health actors (e.g., education, data sciences, engineering) to the relationship between policy and health. Despite calls for integrating health equity across sectors and raising awareness among the public, there were few studies available examining how to advance health equity at an institutional or societal level.

Most articles revealed some degree of concern about the implications of current socio-political trends for health equity work. Among these cautions were concerns about the dominant discourse of bio-behaviourism [42, 45] and the values underlying a broader political economy that elevate the policy priorities which clash with policy environments demonstrating promising progress for health equity [44, 45]. In one synthesis review, for example, neoliberal reforms (e.g., commercialization, internationalization, privilegeing of individual versus collective success) were described as playing a deterministic role in the kinds of research made permissible within the institutional structure of academia, whether by way of appealing to funders or tenure and promotion committees [28]. In combination with a historical academic aversion to advocacy, one scoping review [24] found that an environment of market-driven or austerity-focused university reforms could profoundly restrict academic engagement in health equity work. These cautions pointed to the subtle and far-reaching influences of normative worldviews in creating systems and structures that can constrain the acceptability or legitimacy of health equity work.

## Discussion

Health equity “means all people (individuals, groups and communities) have a fair chance to reach their full health potential and are not disadvantaged by social, economic and environmental conditions” [56]. Yet, social gradients remain deterministic of systematic differences in health and health outcomes both within and between countries [57, 58]. And despite many international declarations of health equity intentions [e.g., 59, 60], little work in this field focuses on interventions aimed at understanding or changing these conditions [14]. This critical interpretive synthesis illuminated evidence about how to enhance such efforts. We provided a schema of four practical, feasible practices for evidence-informed action that may contribute to greater uptake of health equity and highlights opportunities for scholarship, leadership and practice. The practices identified in this review could be applied across a wide range of settings and at different levels (e.g., individually, as teams, and as organizations). They can be informative for anyone whose work is directly or indirectly relevant for health equity, including and extending beyond people working within public health and healthcare systems.

The promising practice of *(re) structuring systems* emphasized the relevance of challenging hierarchical organizational structures and optimizing horizontal partnerships to more effectively capture and implement equity-oriented practices. Our findings support efforts by others to draw attention to healthcare systems, and the governance mechanisms embedded within them, as important sites for intervention on structural determinants of health [61, 62]. Yet, given that much of the action needed to act on these structural determinants of health falls outside the health sector, few examples of cross-sector partnerships or action were found in this synthesis. Research on the role of social power and hierarchy in systems has largely focussed on human resources, management and staff experiences [63]. Yet, class, race, and social power dynamics that exist within institutions can directly and differentially affect people’s access to resources and medical care [64]. Practices identified in this CIS support intentionally developing governance in systems settings (e.g., health, education, and justice systems), with explicit power analysis serving as a mechanism to identify and respond to structural issues of equity. Our findings reinforce the importance of equity-centred healthcare interventions that recognize the need for structural shifts, particularly related to building capacity to identify, assess, and mitigate issues of power. Further, these results demonstrate the need for explicit consideration of how the distribution of power influences health equity work.

Ways of *working relationally* encompassed prioritizing relationship-building and doing research in partnership as methods and accountability mechanisms to promote and achieve health equity. This practice highlighted the need to position research and researchers as one of many partners needed to advance evidence and equity-informed action. Acting as partners rather than independent investigators, invites the adoption methodologies and approaches that explicitly anticipate differences and recognize context in the design, implementation and evaluation of health equity work [65, 66]. Further evaluation of *how* to effectively and meaningfully include a diversity of actors is required. In addition, taking stock of the parameters of inclusion and analysis of social factors that such tools are intended to account for (e.g. gender, class, immigration status) will help researchers and practioners alike continue to expand their efforts towards inclusion. Fostering connectedness, across sectors/disciplines, also enables greater continuity and sustainability in equity initiatives and enable actors to navigate complex systems. This finding reinforces other movements calling for greater authenticity in engagement efforts [61, 62, 67–69]. Again, working relationally is only as promising as it is attuned to assessing and mitigating power.

Promising *research practices* included approaches to inquiry that address complexity by showing sensitivity to socio-political contexts and histories; create spaces for dialogic-relational methods that can catalyze broader coalitions able to affect political action; and address data gaps while demonstrating greater awareness of the implications of constructing indicators and populations in particular ways. Although these findings point to gaps in the sufficiency and comprehensiveness of indicators used in this field, a larger problem is the role of epistemological power—of who gets to decide what counts, what is meansured, and how it is measured. These gaps are not value-neutral, but rather extend from often unarticulated assumptions about what consititutes legitimate ‘data’, ‘evidence’, ‘knowledge’, and ‘expertise’ [70–74]. Indeed, the exclusion of issues (and voices) of those most affected by the unfair global burden of disease has been at the centre of international calls—and a legitimizing argument for expanding global health research investments—for decades [75]. These issues, along with who is included and how they are included, are also issues of governance that, in research, are often afforded far less attention than they warrant.

Embracing complexity and intersecting inequities in the identification of indicators and in data analysis could functionally serve to resist research proclivity for reductionism and bio-behaviourism [70, 76]. Given the role of administrative and clinical data sets gathered and maintained within healthcare systems themselves, it seems an important way of doing research is to actively engage with these systems to advance a more comprehensive consideration of health in the kinds of data that are deemed important enough to collect and monitor. These findings are an invitation to boldy reimagine the mechanisms through which we think about, monitor and report inequities, with particularly exciting possibilities through listening to/opening dialogue that unpacks the ways that normative assumptions about data systems are shaping (and limiting) our capacity to respond to issues of health equity.

Promising *knowledge translation practices* validated the importance of integrated approaches [65, 77], with a particular focus on thinking through who is included in the conversations, and how to substantively contribute to public discourses that may hinder a true commitment to social justice. Many of the promising practices draw attention to issues of governance, which (ideally) focus on the processes through which different sectors and actors deliberate and guide responses to complex public issues [78]. Integrated knowledge translation, with its processes of collaborative articulation of problems, priority setting, gathering and refining evidence, and testing and learning from interventions in context offers strategies to promote *evidence-informed* governance processes [79]. Issues of inclusion, however, are not always addressed by the adoption of an integrated KT approach: they require deliberate effort to identify, examine, and mitigate power imbalances that elevate the concerns and perspectives of some groups over others. This implies a need to consider these promising practices as contingent on embracing their inter-dependence. For example, though our review offers evidence to support the use of integrated approaches, using these approaches without explicit efforts to mitigate the power implications of class and race would present legitimate risks of doing nothing to advance health equity or, worse, doing harm by masking or perpetuating inequities.

### Limitations and strengths

This CIS aimed to critically synthesize promising practices from empirical literature about KWA for health inequities. A key strength of this methodology was that it allowed the identification of emerging evidence through a qualitative analysis of studies demonstrating promise by virtue of being aligned with evidence about causes of inequities. The search terms relied on authors’ use of knowledge translation (or similar) language. We did not include grey or popular literature, nor was it within our scope to explore literature from education, housing, environmental or other sectors. These present important opportunities for future research. There are likely valuable insights available in the broader health equity, social justice, activism, and critical social sciences literature that warrant attention. Articles included in this review were limited to the eight-year period following publication of the World Health Orgnization’s 2008 Commission on Social Determinants of Health [4] report. It is likely that publications from 2017 onward would extend our understanding of the promising practices identified here. Further, articles’ lack of attention to epistemological foundations limited our capacity to examine asssumptions, values, and orientations toward social justice.

## Conclusion

Despite a long-established and robust foundation of evidence about what causes inequities, they persist. Monitoring their stagnance over time will do little to foster health equity. Choosing to embrace a language of *equity* in global or public health implicates a value judgement of health inequities as *unjust*. Ethics in public and global health, therefore, rely on models of distributive or social justice [80, 81] that privilege health as public good and/or human right [82]. Our findings suggest a need for capacity to recognizine how societal structures, including dominant social values such as individualism and bio-behaviourism [83, 84], can promote actions that are directly in conflict with the evidence about root causes of health inequities. Ultimately, our findings offer a set of daily tools to support people to engage in a participatory, democratic exercise that raises collective awareness about the equity options available in any given situation. Moving forward, this CIS creates opportuntieis in KT science to test and refine these promising practices, with particualar attention to the mechanisms by which people in different settings can be supported to integrate them into their daily work. By critically reflecting on dominant discourses and assumptions, and mobilizing political will from a more informed and transparent democratic exercise, knowledge to action for health equity can be achieved.

## Supplementary information

**Additional file 1: Table S1.** Data Extraction and Assessment Summary, Literature Reviews.

**Additional file 2: Table S2.** Data Extraction and Assessment Summary, Research Studies.

**Additional file 3: Table S3.** Promising Practices for (Re) Structuring Systems.

**Additional file 4: Table S4.** Promising Practices for Working Relationally.

**Additional file 5: Table S5.** Promising Practices for Doing Research.

**Additional file 6: Table S6.** Promising Practices for Knowledge Translation.

## Data Availability

All data generated or analysed during this study are included or cited in this published article [and its Additional files].
