# Supplementary material for: Connecting knowledge with action for health equity: a critical interpretive synthesis of promising practices
Source: Int J Equity Health. 2019 Dec 26;18:202. doi: 10.1186/s12939-019-1108-x (PMC6933619; doi:10.1186/s12939-019-1108-x)
Supplement: Supplementary file 1 — Additional file 1: Table S1. Data Extraction and Assessment Summary, Literature Reviews. [file 12939_2019_1108_MOESM1_ESM.docx]

**Supplementary Table 1. Data Extraction and Assessment Summary, Literature Reviews**

| **Authors (Year)**  **Discipline, Location*** | **Title** | **Study Purpose** | **Methods** | **Practices Examined or Derived** | **Assessment of Articles** | | | |
| --- | --- | --- | --- | --- | --- | --- | --- | --- |
|  |  |  |  |  | **Orientation to Root Causes** | **Clarity/Quality Assessment**  **AO + Des + Met + D + An**  **Comments** | **Clarity Quality**  **Score** | |
| Andermann (2016)  Medicine, Canada | Taking action on the social determinants of health in clinical practice: A framework for health professionals | To provide "concrete actions" that clinicians can use in their daily practice to help address SDOH (p. E474). | Realist review | Individual, neighborhood, and community-level health equity practices of practitioners (especially physicians) | Interrupt | AO(0) + Des(0) + Met(1) + D(1) + An(0)  PRISMA guidelines not followed | | 2 |
| Carey, Crammond, & Keast (2014)  Population Health, Australia | Creating change in government to address the social determinants of health: How can efforts be improved? | “to identify lessons from the exiting [sic] body of evidence JUG [joined-up government], which can help strengthen IPIs currently being implemented, through a meta-analysis of joined-up government initiatives” (p. 5). | Meta-synthesis of policy research | Systems-level interventions for leveraging health equity, using Johnston's intervention level framework and Meadow's 12 places to intervene | Illuminate | AO(2) + Des(2) + Met(1) + D(2) + An(1)  PRISMA guidelines followed | | 8 |
| Chircop, Basset, & Taylor (2015)  Nursing, Canada | Evidence on how to practice intersectoral collaboration for health equity: A scoping review | “to generate insight into the current scientiﬁc literature to scope out the extent of peer-reviewed publications on intersectoral collaboration for public policy toward health equity, and to identify gaps in the literature about evidence-based approaches to intersectoral collaboration practices for health equity-  oriented policy action” (p. 180). | Scoping review | Intersectoral collaboration as a practice for policy action for health equity | Illuminate | AO(2) + Des(2) + Met(1) + D(2) + An(1)  PRISMA guidelines not followed, but scoping review methodology clearly described | | 8 |
| Cohen & Marshall  (2017)  Nursing, Canada | Does public health advocacy seek to redress health inequities? A scoping review | “to obtain an overview of the literature related to public health advocacy, with a particular interest in the extent to which this literature addresses the goal of reducing the social, environmental and structural causes of health and social inequities” (p. 310) | Scoping review | Public health advocacy for reducing the social, environmental and structural causes of health and social inequities | Illuminate | AO(1) + Des(2) + Met(2) + D(2) + An(2)  Advocacy not defined  PRISMA guidelines followed | | 9 |
| Davison, Ndumbe-Eyoh, & Clement  (2015)  Public Health, Canada | Critical examination of knowledge-to- action models and implications for promoting health equity | “to identify existing knowledge to action models or frameworks and critically examine a promising subset of them as to their utility for promoting or supporting health equity” (p. 2). | Scoping review | Knowledge-to-action frameworks that describe ways to bridge the *know-do gap* | Acknowledge | AO(2) + Des(0) + Met(2) + D(2) + An(2)  PRISMA guidelines not followed | | 8 |
| Farrer et al. (2015)  Public Health, Belgium | Advocacy for health equity: A synthesis review | "to synthesize the evidence in the academic and gray literature and to provide a body of knowledge for advocates to draw on to inform their efforts." (p. 392) | Critical interpretive synthesis | Advocacy, defined *as a deliberate attempt to influence decision makers and other stakeholders to support or implement policies that contribute to improving health equity using evidence* | Illuminate | AO(2) + Des(2) + Met(2) + D(2) + An(1)  PRISMA guidelines followed | | 9 |
| Ndumbe-Eyoh & Moffatt  (2013)  Health Sciences, Canada | Intersectoral action for health equity: A rapid systematic review | “to examine the impact and  effectiveness of intersectoral action as a public health practice for health equity through action on the SDH” (p. 2) | Rapid systematic review | Intersectoral interventions, policies and programs, undertaken by the public health sector in collaboration with governmental and non-governmental sectors outside of health | Illuminate | AO(2) + Des(2) + Met(2) + D(2) + An(1)  PRISMA guidelines followed | | 9 |
| Newman et al. (2015)  Geography, Australia | Addressing social determinants of health inequities through settings: A rapid review | To provide “a rapid review of what settings-based health promotion approaches are effective in addressing the social determinants of health inequities” (p. 127). | Rapid review | Work in settings that has reduced, or shown promise in reducing, health inequities; settings approaches that address social determinants of health; and policy and programme work in settings | Illuminate | AO(2) + Des(2) + Met (1)+ D(2) + An(0)  PRISMA guidelines not followed; analytical processes not described | | 7 |
| Shareck, Frohlich, & Poland  (2013)  Social Medicine, Canada | Reducing social inequities in health through settings-related interventions: A conceptual framework | To understand how a settings approach can inform action to reduce social inequities in health by identifying challenges and “proposing a ‘settings praxis’ to help overcome them and inform an innovative, equity-focused use of the settings” (p. 40). | Scoping review | How to avoid exacerbating inequities through settings approaches; what (and how to apply) elements of a settings approach specifically focus on reducing social inequities in health | Illuminate | AO(2)+ Des(0) + Met(1) + D(2) + An(1)  Scoping review methodology not described; PRISMA guidelines not followed | | 6 |
| Weiler et al. (2015)  Sociology, Canada | Food sovereignty, food security and health equity: A meta-narrative mapping exercise | “1) to map key narratives from scholarly research on intersections between food security and health equity and (2) to identify evidence of how food sovereignty interventions can be implemented to promote health equity” (p. 1081). | Narrative synthesis | How to apply food sovereignty principles to health equity research and practice, which emphasizes communities' power to democratically manage food systems resources and trade | Illuminate | AO(2) + Des(2) + Met(1) + D(2) + An(2)  Clear analytic framework provided;  PRSIMA guidelines followed (although flow chart not provided) | | 9 |

*Discipline and location reported for primary author
