# Supplementary material for: Connecting knowledge with action for health equity: a critical interpretive synthesis of promising practices
Source: Int J Equity Health. 2019 Dec 26;18:202. doi: 10.1186/s12939-019-1108-x (PMC6933619; doi:10.1186/s12939-019-1108-x)
Supplement: Supplementary file 2 — Additional file 2: Table S2. Data Extraction and Assessment Summary, Research Studies. [file 12939_2019_1108_MOESM2_ESM.docx]

**Supplementary Table 2. Data Extraction and Assessment Summary, Research Studies**

| **Authors (Year)**  **Discipline, Location*** | **Title** | **Study Purpose** | **Methods** | **Practices Examined or Derived** | **Assessment of Articles** | | | |
| --- | --- | --- | --- | --- | --- | --- | --- | --- |
|  |  |  |  |  | **Orientation to Root Causes** | **Clarity/Quality Assessment**  **AO + Des + Met + D + An**  **Comments** | **Clarity Quality**  **Score** | |
| Baum et al. (2010)  Public Health, Australia | Can a regional government's social inclusion initiative contribute to the quest for health equity? | To examine “the evidence to determine if a social inclusion initiative is a useful aspect of government action to reduce health inequity” (p. 475). | Rapid appraisal using case studies | Social inclusion initiative  Apply the WHO Social Exclusion Knowledge Network framework | Illuminate | AO(1) + Des(1) + Met(1) + D(1) + An(2)  Document review + policy analysis + key informant interviews (n=10) | | 5 |
| Baum et al. (2013)  Public Health, Australia | "Never mind the logic, give me the numbers": Former Australian health ministers' perspectives on the social determinants of health | To report “empirical information relevant to theoretical debates about political and other factors inﬂuencing translation of evidence into policy. It reports on the views of twenty former health ministers about policy opportunities during their tenure to address SDH and health inequities” (p. 139). | Qualitative | Policy windows for action on SDH  Apply Kingdon’s Policy theory | Illuminate | AO(1) + Des(0) + Met(1) + D(1) + An(1)  Interviews with former health ministers (n=20)  No specific qualitative approach named | | 4 |
| Blanchard et al. (2013)  Health Promotion, France | Improving policy and practice to promote equity and social justice: A qualitative comparative analysis building on key learnings from a twinning exchange between England and the US | “to share and develop a better understanding of strategies for actions that can effectively address the SDH and social injustice” (p.46). | Qualitative | Learning exchanges used as a means to identify and examine lessons learnt, drivers, factors for success and strategies that work for action on SDH | Illuminate | AO(1) + Des(1) + Met(2) + D(1) + An(1)  Questionnaires (n=16 organizations)  No specific qualitative approach named, state use ‘thematic analysis’ in analysis | | 6 |
| Borde, Akerman, & Pelligrini  (2014)  Public Health, Brasil | Mapping of capacities for research on health and its social determinants in Brazil | “to contribute to the identification of capacities for research on health and its social determinants in Brazil by mapping research activities and the scope of SDH research as well as research system structures, policies and networks defining research on health and its social determinants in Brazil, focusing on the developments since the establishment of the World Health Organization Commission on SDH (CSDH) in 2005” (p. 2082). | Mapping study | Health research systems and capacity for SDH research | Illuminate | AO(1) + Des(0) + Met(2) + D(1) + An(1)  Literature reviews + semi-structured key informant interviews + database (knowledge translation platforms) consultations  No specific mapping methodology described | | 5 |
| Brassolotto et al. (2014)  Health Policy, Canada | Epistemological barriers to addressing the social determinants of health among public health professionals in Ontario, Canada | “to examine our assumption that there might be epistemological challenges to PHUs applying [social determinants of health] concepts… [and] to understand the worldviews of public health ofﬁcials concerning these issues” (p. 2). | Qualitative interviews | Daily public health work | Illuminate | AO(1) + Des(0) + Met(1) + D(2) + An(2)  No specific qualitative approach named; orientation to root causes of health inequities well described | | 6 |
| Cacari-Stone et al. (2014)  Public Health, United States of America | The promise of community-based participatory research for health equity: A conceptual model for bridging evidence with policy | To explore how community-based participatory research (CBPR) responds to two knowledge-to-action challenges: “1) the gap between scientiﬁc evidence and policy action based on evidence, and (2) the difﬁculty of mobilizing civic engagement for policymaking in the United States” (p. 1615). | Descriptive case study | Community-based participatory research practices  Derive a framework linking CBPR and policy making | Interrupt | AO(1) + Des(0) + Met(0) + D(1) + An(1)  No specific qualitative approach named | | 3 |
| Carey & Crammond (2015)  Epidemiology & Public Health, Australia | Systems change for the social determinants of health | To review evidence on joined-up approaches, illuminating ‘system leverage points’ and how action for change can be effective in complex systems that determine health outcomes. | Policy analysis | Systems-level approaches for leveraging action on causes of health inequities  Analytical framework includes an adapted version of Meadow’s ’12 places to intervene in a system’ | Illuminate | AO(1) + Des(2) + Met(2) + D(2) + An(2) | | 9 |
| deAndre et al. (2015)  Medicine, Brasil | Social determinants of health, universal health coverage, and sustainable development: Case studies from Latin American countries | To “assess the experiences in the design and implementation at national scale social programmes underpinned by intersectoral action and social participation aimed at addressing social determinants of health, improving health, and reducing health inequities” (p. 1344). | Case series* | Health systems collaboration with other sectors to address upstream determinants of health; universal health coverage | Interrupt | AO(0) + Des(1) + Met(1) + D(1) + An(2)  Document review + secondary analysis of public health outcomes data (n=4 cases: Brasil, Chile, Columbia, and Cuba)  Strong analysis of sociopolitical and historical contexts | | 5 |
| Estey et al. (2010)  Knowledge Translation, Canada | Thinking about aboriginal KT: learning from the Network Environments for Aboriginal Health Research British Columbia (NEARBC) | “to highlight the complexity of Aboriginal KT (a shorthand used by the authors to refer to KT in Aboriginal health research contexts), but also areas for action” (p. 83). | Exploratory case study | Indigenous approaches in knowledge translation | Illuminate | AO(2) + Des(1) + Met(1) + D(2) + An(1)  Interviews with people involved in the Network Environments for Aboriginal Research British Columbia  (n=10) | | 7 |
| Gore & Kothari (2012)  Health Sciences, Canada | Social determinants of health in Canada: Are healthy living initiatives there yet? A policy analysis | “to evaluate healthy living initiatives in  BC and ON that focus on healthy eating and physical activity based on their approach to the social determinants of health and health inequities” (p. 3). | Policy analysis | Healthy living initiatives  Derive an analytical framework to assess policies for type of initiative (life-style, environment, or structural) and mechanism (direct program, blueprint, or building block) | Illuminate | AO(2) + Des(2) + Met(1) + D(2) + An(2)  Searched documents, website, database, and health organizations to identify initiatives in 2 Canadian provinces (n=60 Ontario initiatives; n=61 British Columbia initiatives) | | 9 |
| Grundy et al. 2014  Global Health, Cambodia | Improving average health and persisting health inequities: Towards a justice and fairness platform for health policy making in Asia | To “describe the existing trends in inequity reduction” in eight Asian countries (Bangladesh, Cambodia, India, Indonesia, Nepal, Pakistan, Phillipines, Vietnam) …“and then to consider the implications of these findings for the practice of health policy making at national and global level” (p. 875). | Case Comparison | Wealth re-distribution and universal access policies | Illuminate | AO(1) + Des(1) + Met(1) + D(1) + An(1)  Literature review + secondary analysis of Demographic Health Survey Data  No specific methodological approach named | | 5 |
| Kirst et al. (2017)  Psychology, Canada | Addressing health inequities in Ontario, Canada: What solutions do the public support? | To “examine the relationship between how the public attribute health inequities and support for targeted vs. broader health equity interventions” (p. 2). | Survey study with multivariate analysis | Public knowledge and opinion about health equity; call for messaging that connects health inequities with distribution of power and privilege between rich and poor | Illuminate | AO(2) + Des(2) + Met(2) + D(2) + An(2)  Telephone surveys (n=2006) | | 10 |
| Knight (2014)  Public Policy, USA | Shifting public health practice to advance health equity: Recommendations from experts and community leaders | To “build on the *Unnatural Causes* campaign by gathering and disseminating recommendations about public health strategies for achieving health equity” (p. 189). | Qualitative | Documentary as means of raising public awareness of nature and context of health inequities | Illuminate | AO(1) + Des(1) + Met(1) + D(2) + An(1)  Interviews  (n=29) | | 6 |
| Labonté et al. (2014)  Population Health, Canada | Is the Alma Ata vision of comprehensive primary health care viable? Findings from an international project | To explore how mentored research teams using an integrated knowledge translation approach (teams included triads of junior + senior researchers and research users) could integrate health equity work through primary health care initiatives in 20 teams from multiple countries | Case series* | Action on structural and SDH through comprehensive primary healthcare | Illuminate | AO(2) + Des(1) + Met(1) + D(2) + An(1)  Synthesis of findings from 20 different studies using a common mentored and integrated knowledge translation approach; methodological foundations for synthesis not described | | 7 |
| McPherson et al. (2016)  Nursing, Canada | Swimming against the tide: A Canadian qualitative study examining the implementation of a province-wide public health initiative to address health equity | To “examine the strategy of developing and implementing equity-focused positions to improve public health organizational capacity to act on SDH and advance health equity” (p. 3). | Descriptive qualitative case study | Public health nursing practices | Interrupt | AO(2) + Des(2) + Met(2) + D(2) + An(2)  Interviews  (n=42) + document review (n=226) | | 10 |
| Mtenga, Masanja, & Mamdani (2016)  Social Science, Tanzania | Strengthening national capacities for researching on social determinants of health (SDH) towards informing and addressing health inequities in Tanzania | To explore “the SDH landscape in Tanzania. Specifically, the conceptualisation, nature, extent and reach of SDH research, supporting national systems and processes for SDH research” (p. 3). | Research systems mapping | Cultivating structure and capacity for health research systems  Some gaps in methodological clarity, but strong integration of SDH capacity-building framework and comprehensive discussion of methods (process) and results | Illuminate | AO(1) + Des(1) + Met(2) + D(2) + An(1)  In-depth stakeholder interviews (n=34) + policy analysis + SDH research outputs (published) from 2005 onward | | 7 |
| Murphy et al. (2015)  Health Sciences, Canada | Making a commitment to ethics in global health research partnerships: A practical tool to support ethical practice | To create processes and tools for enabling “respectful, mutually beneficial, and effective North–South research partnerships” (p.141). | iKT/PAR | Equity-promoting practices for research partnerships, especially in global health research | Interrupt | AO(2) + Des(1) + Met(1) + D(2) + An(2)  Engaged consultation + dialogue using local case studies  Consultations held in South Asia, Africa, & Latin America, n≈30 per site | | 8 |
| Povall et al. (2014)  Public Health Policy, UK | Health equity impact assessment | To “determine whether or not a new HEIA [health equity impact assessment] methodology is needed to examine the health equity impacts of global, regional, national and local financial and public policies" (p. 622). | Mixed methods* | Health equity impact assessment methodologies and equity-attunement | Illuminate | AO(2) + Des(2) + Met(1) + D(1) + An(2)  Scoping review + interviews (n=14) + workshops (n=19) | | 8 |
| Raphael & Brassolotto (2015)  Health Policy, Canada | Understanding action on the social determinants of health: A critical realist analysis of in-depth interviews with staff of nine Ontario public health units | To “consider the factors that shape local PHU action on the SDH through a critical realist analysis" (p. 1) by “elucidating the existing societal structures and powers that enable SDH-related activities and identifying factors that either facilitate or prevent the activation of these structures and powers. We also consider how institutional factors interact with personal characteristics of MOHs and features of local jurisdictions to shape the form of SDH activities” (p. 3). | Critical realist approach | Leadership practices and perspectives of public health leaders and workers | Illuminate | AO(2) + Des(2) + Met(2) + D(2) + An(2)  Document review  + interviews (n=18) | | 10 |
| Raphael, Brassolotto, & Baldeo  (2014)  Health Policy, Canada | Ideological and organizational components of differing public health strategies for addressing the social determinants of health | To “investigate how individual characteristics of Medical Officers of Health (MOH) and unit staff (e.g. background training, personal experiences and understandings of the SDH), community features (e.g. urban versus rural, political climate and governance structures) and organizational features (e.g. central versus devolved SDH structures, leader- ship and training) account for these differences in PHUs’ SDH activities” (p. 856). | Qualitative | Ideological and organizational characteristics relationship to the type of SDH work carried out in public health units  Findings suggest a need for more structural approaches | Illuminate | AO(1) + Des(1) + Met(1) + D(2) + An(1)  Document analysis + interviews (n=18) | | 6 |
| Tolhurst et al. (2012)  International Health, UK | Intersectionality and gender mainstreaming in international health: Using a feminist participatory action research process to analyse voices and debates from the global south and north | To “contribute to the debate on the implications of [the] failure for future action for gender equity in health” through “productive dialogue” that brings “together the voices of disparate actors, first heard in a series of four seminars held during 2008 and 2009, involving almost 200 participants from 15 different country contexts” (p. 1826). | Feminist  participator-y action research | Research, policy, and knowledge translation practices for promoting gender equity | Interrupt | AO(2) + Des(2) + Met(1) + D(2) + An(2)  Dialogues (n=200+) | | 9 |
| Young (2011)  Health Sciences, Australia | Exploring discourses of equity, social justice and social determinants in Australian health care policy and planning documents | To assess how “social determinants understanding [is] demonstrated in Australian health policy documents,” questioning if an SDH or alternate framework prevails (p. 369). | Discourse analysis | Use of SDH language and discourses in policy writing at “micro (word searching), meso (documentary overview and reﬂection) and macro (sociopolitical reﬂection) levels” (p. 370) | Illuminate | AO(2) + Des(2) + Met(2) + D(2) + An(2)  Analysis of policy documents (n=8) | | 10 |

*Discipline and location reported for primary author
