# Supplementary material for: Connecting knowledge with action for health equity: a critical interpretive synthesis of promising practices
Source: Int J Equity Health. 2019 Dec 26;18:202. doi: 10.1186/s12939-019-1108-x (PMC6933619; doi:10.1186/s12939-019-1108-x)
Supplement: Supplementary file 3 — Additional file 3: Table S3. Promising Practices for (Re) Structuring Systems. [file 12939_2019_1108_MOESM3_ESM.docx]

**Supplementary Table 3. Promising Practices for (Re)Structuring Systems**

| **Promising Practices** | **How to do it** | **Citations for supporting evidence (First Author, Year)** |
| --- | --- | --- |
| Embed a policy of health equity at systems-levels | Integrate human rights obligations with KT messaging (e.g., health inequities data) to advance high-level integration of health equity. | Blanchard 2013, Cohen 2017, Davison 2015, Farrer 2015, Labonté 2014, Mtenga 2016, Newman 2015, Povall 2013, Shareck 2013. |
|  | Integrate a commitment to health equity as a foundational principle and/or strategic goal in governance structures, such as governments (municipal, regional, provincial/state, federal), public agencies (e.g., healthcare systems, educational systems), and professional bodies. | Borde 2014, Brassolotto 2013, Cohen 2017, Knight 2014, Labonté 2014,; McPherson 2016, Raphael 2014, Raphael 2015, Weiler 2015. |
| Integrate (defragment) governance mechanisms  *This practice is particularly promising when also using promising practices for working relationally.* | Identify, assess, and adapt governance processes and bodies involved in making decisions about who is involved, what is to be achieved, and how it is to be implemented (Kelaher, 2014) for health equity (and equity-related) work, striving for transparency. | Baum 2010, Carey 2014, Chircop 2014, de Andrande 2015, Labonté 2014, McPherson 2016, Newman 2015, Raphael 2015, Shareck 2013. |
|  | Use tools that enable integrated (i.e., cross-sector and discipline) governance, such as equity-sensitive health impact assessments, health-in-all policies, or joined-up or whole-of-government approaches to address health equity issues and needs at the population level. | Baum 2010, Brassolotto 2013, Carey 2014, Chircop 2015, Knight 2014, Newman 2015, Povall 2014, Raphael 2015, Shareck 2013. |
|  | Actively engage education sectors in efforts to embed health equity in policy, with particular attention to raising public awareness of the relationship between political economy and social justice in school settings (i.e., with children and youth) and enhancing both knowledge and capacity through the education of health professionals (nurses, physicians, allied health) and social workers. | Andermann 2016, Cohen 2017, de Andrande 2015, Farrer 2015, Gore 2012, Newman 2015, Raphael 2015, Weiler 2015. |
| Strategically navigate bureaucratic hierarchies | Be attentive to bureaucratic culture and norms and issues leadership, accountability, influence, and authority when embedding health equity work or dedicated roles. | Brassolotto 2013, Carey 2014, Chircop 2014, McPherson 2016, Raphael 2014, Shareck 2013, Young 2011. |
|  | Align health equity agendas with structural mechanisms to enable action. | Brassolotto 2013, Carey 2014, McPherson 2016, Newman 2015. |
|  | Cultivate health equity champions and leaders. | Blanchard 2013, Brassolotto 2013, Cacari-Stone 2014, Carey 2014, Davison 2015, de Andrande 2015, Labonté 2014, Raphael 2014, Raphael 2015. |
