# Supplementary material for: Connecting knowledge with action for health equity: a critical interpretive synthesis of promising practices
Source: Int J Equity Health. 2019 Dec 26;18:202. doi: 10.1186/s12939-019-1108-x (PMC6933619; doi:10.1186/s12939-019-1108-x)
Supplement: Supplementary file 4 — Additional file 4: Table S4. Promising Practices for Working Relationally. [file 12939_2019_1108_MOESM4_ESM.docx]

**Supplementary Table 4. Promising Practices for Working Relationally**

| **Promising Practices** | **How to do it** | | **Citations for supporting evidence**  **(First Author, Year)** | |
| --- | --- | --- | --- | --- |
| Foster connections and connectedness to enable more responsive, complexity-sensitive health equity work | Adopt transdisciplinary, cross-sector approaches to health equity work across multiple levels (i.e., local/municipal, regional, national, and international/global), maintaining an upstream-structural focus. | Baum 2010, Baum 2013, Blanchard 2013, Borde 2014, Brassolotto 2013, Chircop 2015, Cohen 2017, Davison 2015, de Andrande 2015, Estey 2010, Farrer 2015, Gore 2012, Kirst 2017, Labonté 2014, McPherson 2016, Mtenga 2016, Ndumbe-Eyoh 2013, Newman 2015, Raphael 2015, Shareck 2013, Weiler 2015 | |  |
|  | Identify and examine underlying assumptions and discourses, with attention to how they shape the ways in which health inequities are framed and responded to (especially taken-for-granted assumptions underlying dominant discourses, such as neoliberalism, individualism, and bio-behaviourism). | Brassolotto 2013, Farrer 2015, Grundy 2014, Mtenga 2016, Raphael 2015, Weiler 2015, Young 2011 | |  |
|  | Cultivate networks and supported learning communities as platforms for responsive collaboration and learning that can open new possibilities for doing structural health equity work. | Andermann 2016, Blanchard 2013, Labonté 2014, McPherson 2016, Mtenga 2016, Raphael 2014, Raphael 2015 | |  |
|  | Take advantage of windows of opportunity on issues that, though narrow, could be creatively leveraged to respond to shared social and structural determinants of health. | Blanchard 2013, Farrer 2015, Gore 2012, Knight 2014, Labonté 2014, McPherson 2016, Tolhurst 2012 | |  |
|  | Create a culture of collective responsibility and action; fostering a willingness to share roles and expertise is encouraged along with responsibilities and collective action. | Davison 2015, Estey 2010, Knight 2014, Wieler 2015 | |  |
| Foster inclusion of non-academic partners, with particular attention to those who may be historically excluded due to race/ethnicity, culture, gender, sexual orientation, Indigeneity, ableness, or other –isms | Actively assess for and mitigate inequities of why, who, and how particular people or groups are engaged in health equity work. | Murphy 2015, Labonté 2014, de Andrande 2015 | |  |
|  | Create inclusive priority-setting and integrated KT processes. | Cacari-Stone 2014, Gore 2012, Murphy 2015, Knight 2014, Borde 2014, Labonté 2014, Mtenga 2016 | |  |
|  | Include ‘outside-the-box,’ non-scientific and non-health actors, such as the media, social movements, and other actors that appeal to public sentiment and political will in health equity work. | Borde 2014, Brassolotto 2013, Cacari-Stone 2014, Carey 2014, Cohen 2013, de Andrande 2015, Farrer 2015, Gore 2012, Kirst 2017, Knight 2014, McPherson 2016, Newman 2015, Raphael 2014, Raphael 2015, Weiler 2015 | |  |
| Mitigate power imbalances | Adopt a standard practice of critically reflecting on, assessing, and mitigating the distribution of power among all actors engaged in (and affected by) all health equity work. | Blanchard 2013, Murphy 2015, Raphael 2014, Shareck 2013, Tolhurst 2012, Young 2011 | |  |
|  | Leverage power and resources to mitigate historical and contextual power imbalances. | Andermann 2016, Blanchard 2013, Labonté 2014, Murphy 2015 | |  |
|  | Use equity-sensitive tools to assess, monitor, and purposively elevate voices that have been historically silenced or subjected to structural power inequities in both health-equity responsive governance mechanisms and research processes. | de Andrande 2015, Estey 2010, Knight 2014, Murphy 2015 | |  |
