# Supplementary material for: Connecting knowledge with action for health equity: a critical interpretive synthesis of promising practices
Source: Int J Equity Health. 2019 Dec 26;18:202. doi: 10.1186/s12939-019-1108-x (PMC6933619; doi:10.1186/s12939-019-1108-x)
Supplement: Supplementary file 5 — Additional file 5: Table S5. Promising Practices for Doing Research. [file 12939_2019_1108_MOESM5_ESM.docx]

**Supplementary Table 5. Promising Practices for Doing Research**

| **Promising Practices** | | **How to do it** | | **Citations for supporting evidence**  **(First Author, Year)** | |
| --- | --- | --- | --- | --- | --- |
| Design for context, complexity | Consider informing research with foundational theories that are compatible with complexity, such as complexity theory, critical realism, intersectionality, and critical theories. | | Brassolotto 2013, Newman 2015, Mtenga 2016, Povall 2014, Raphael 2015, Tolhurst 2012, Weiler 2015 | |  |
|  | Adopt context-responsive research designs that acknowledge history and power distribution throughout research and knowledge translation processes. | | Blanchard 2013, Brassolotto 2013, Chircop 2015, Davison 2015, McPherson 2016, Mtenga 2016, Povall 2014 | |  |
|  | Diversify research approaches to explore complex problems, foster inclusion, and spark civic engagement, using multiple and non-traditional modes (e.g., community-based participatory research) of inquiry. | | Borde 2014, Cacari-Stone 2014, Davison 2015, Estey 2010, Shareck 2013, Tolhurst 2012 | |  |
| Use dialogic-relational methods | Consider building team dialogue into research designs as a means of enabling critical reflection about how to operationalize health equity considerations in research and advancing collective understanding and capacity to implement structurally-oriented interventions. | | Labonté 2014, McPherson 2016, Mtenga 2016, Murphy 2015, Tolhurst 2012 | |  |
|  | Integrate inclusive dialogue as a means of refining and mobilizing responses to evidence about health inequities. | | Andermann 2016, Borde 2014, Cacari-Stone 2014, Carey 2014, Estey 2010, Gore 2012, Labonté 2014, Tolhurst 2012, Weiler 2015 | |  |
| Ameliorate gaps in data platforms and indicators | Create reliable, systematically collected data in mechanisms that can serve as responsive feedback loops. | | Borde 2014, Carey 2014, de Adrande 2015, Gore 2012, Povall 2014 | |  |
|  | Expand commonly used set of health inequities indicators to include upstream indicators of health equity, social and structural determinants of health, and power distribution. | | Blanchard 2013, Borde 2014, McPherson 2016, Povall 2014, Tolhurst 2012, Young 2011 | |  |
|  | Make the links between policy decisions (action and inaction) and health clear and compelling, especially between macro-level and health. | | Carey 2014, Grundy 2014, Knight 2014, Mtenga 2016, Weiler 2015, Young 2011 | |  |
|  | Assess and mitigate the impacts of aggregation, particularly in masking social gradients. | | de Andrade 2015, Gore 2012, Grundy 2014, Povall 2014, Tolhurst 2012 | |  |
