# Supplementary material for: Connecting knowledge with action for health equity: a critical interpretive synthesis of promising practices
Source: Int J Equity Health. 2019 Dec 26;18:202. doi: 10.1186/s12939-019-1108-x (PMC6933619; doi:10.1186/s12939-019-1108-x)
Supplement: Supplementary file 6 — Additional file 6: Table S6. Promising Practices for Knowledge Translation. [file 12939_2019_1108_MOESM6_ESM.docx]

**Supplementary Table 6. Promising Practices for Knowledge Translation**

| **Promising Practices** | **How to do it** | | **Citations for supporting evidence**  **(First Author, Year)** | |
| --- | --- | --- | --- | --- |
| Practice integrated approaches | Prioritize processes that include a broad range of research users alongside producers in research in social processes that foster trust and dialogue and are responsive to context and issues of power. | Davison 2015, Estey 2010, Labonté 2014, Murphy 2015 | |  |
|  | Critically reflect upon and strategically respond to political will and political economy. | Baum 2013, Brasolotto 2013, Raphael 2014, Raphael 2015 | |  |
|  | Package evidence in ways that present a concise and compelling story that includes feasible policy options and presents timely, real-life data to policy makers. | Baum 2010, Baum 2013, Cacari-Stone 2014, Farrer 2015 | |  |
| Be creative | Produce non-academic outputs (e.g., documentary, imagery) to share results of research, particularly with the public. | Borde 2014, Cacari-Stone 2014, Cohen 2017, Shareck 2013 | |  |
|  | Use metaphors and other arts-based approaches to curating evidence. | Farrer 2015, Knight 2014, Shareck 2013 | |  |
| Use evocative messages that spark empathy and connection. | Humanize data through use of stories told in the voices of people with lived experience. | Cacari-Stone 2014, Farrer 2015, Gore 2012, Kirst 2017, Povall 2014, Raphael 2014, Raphael 2015 | |  |
|  | Use stories that illuminate the ways in which structural and social power work to create inequities, countering norms of focusing on individuals and behaviours. | Cacari-Stone 2014, Cohen 2017, Farrer 2015, Kirst 2017, Knight 2014, Povall 2014 | |  |
